# Supplementary material for: All-at-once RNA folding with 3D motif prediction framed by evolutionary information
Source: Nat Methods. 2025 Oct 3;22(10):2094–106. doi: 10.1038/s41592-025-02833-w (PMC12510878; doi:10.1038/s41592-025-02833-w)
Supplement: Supplementary file 3 — Source code to run CaCoFold-R3D. Data and results. [file 41592_2025_2833_MOESM3_ESM.gz › supplemental_material/results/CaCoFold-R3D/CaCoFold-R3D_Rfam_vshuffle/RF00020_U5.R2R.sto.pdf]

RF00020_U5

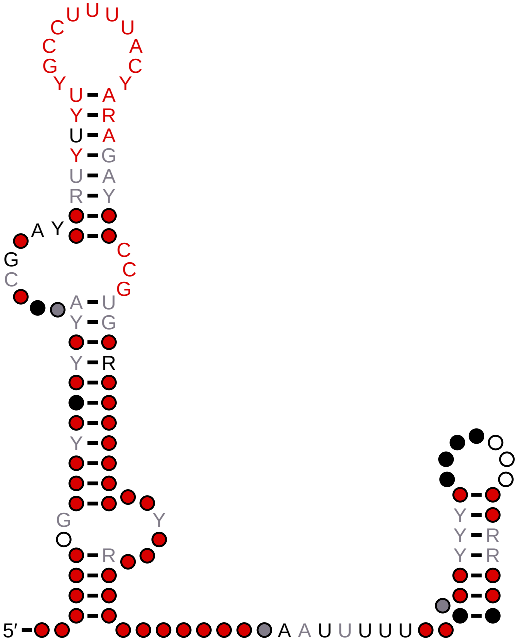

Y G C C U U U U A C Y
U – A
Y – R
U – A
Y – G
U – A
R – Y
A Y
G
C
C C G
A – U
Y – G
Y – R
Y
G
Y
R
5′
A A U U U U U U
Y
Y – R
Y – R
